# Impact of the Phytoestrogen Content of Laboratory Animal Feed on the Gene Expression Profile of the Reproductive System in the Immature Female Rat

**DOI:** 10.1289/ehp.6848

**Published:** 2004-08-16

**Authors:** Jorge M. Naciff, Gary J. Overmann, Suzanne M. Torontali, Gregory J. Carr, Jay P. Tiesman, George P. Daston

**Affiliations:** Miami Valley Laboratories, The Procter and Gamble Company, Cincinnati, Ohio, USA

**Keywords:** 17α-ethynyl estradiol, gene expression profiling, immature rat uterotrophic assay, microarrays, phytoestrogens, rodent diet

## Abstract

The effect of the dietary background of phytoestrogens on the outcome of rodent bioassays used to identify and assess the reproductive hazard of endocrine-disrupting chemicals is controversial. Phytoestrogens, including genistein, daidzein, and coumestrol, are fairly abundant in soybeans and alfalfa, common ingredients of laboratory animal diets. These compounds are weak agonists for the estrogen receptor (ER) and, when administered at sufficient doses, elicit an estrogenic response *in vivo*. In this study, we assessed the potential estrogenic effects of dietary phytoestrogens at the gene expression level, together with traditional biologic end points, using estrogen-responsive tissues of the immature female rat. We compared the gene expression profile of the uterus and ovaries, as a pool, obtained using a uterotrophic assay protocol, from intact prepubertal rats fed a casein-based diet (free from soy and alfalfa) or a regular rodent diet (Purina 5001) containing soy and alfalfa. Estrogenic potency of the phytoestrogen-containing diet was determined by analyzing uterine wet weight gain, luminal epithelial cell height, and gene expression profile in the uterus and ovaries. These were compared with the same parameters evaluated in animals exposed to a low dose of a potent ER agonist [0.1 μg/kg/day 17α-ethynyl estradiol (EE) for 4 days]. Exposure to dietary phytoestrogens or to a low dose of EE did not advance vaginal opening, increase uterine wet weight, or increase luminal epithelial cell height in animals fed either diet. Although there are genes whose expression differs in animals fed the soy/alfalfa-based diet versus the casein diet, those genes are not associated with estrogenic stimulation. The expression of genes well known to be estrogen regulated, such as progesterone receptor, intestinal calcium-binding protein, and complement component 3, is not affected by consumption of the soy/alfalfa-based diet when assessed by microarray or quantitative reverse transcriptase–polymerase chain reaction analysis. Our results indicate that although diet composition has an impact on gene expression in uterus and ovaries, it does not contribute to the effects of an ER agonist.

The effect of the dietary background of phytoestrogens on the outcome of rodent bioassays used to identify and assess the reproductive hazard of endocrine-disrupting chemicals is controversial. Phytoestrogens, including genistein, daidzein, and coumestrol, are fairly abundant in soybeans and alfalfa, common ingredients of laboratory animal diets. In fact, soy and alfalfa are commonly used as protein sources in the manufacture of most rodent diets. Some of these ingredients are known to contain endocrine modulators, such as the phytoestrogens genistein and daidzein (abundant in soybeans and its products) and their respective glycosides (genistin and daidzin), and coumestrol (found in alfalfa). These phytoestrogens are able to bind to both estrogen receptor (ER) isoforms, ER-αand ER-β, *in vitro* (Beck et al. 2003; Casanova et al. 1999). They have a higher affinity for ER-β (Boue et al. 2003), but they activate both ER isoforms, although with less potency than estradiol. Both genistein and daidzein have much weaker affinities than does 17β-estradiol for the rat ERs: genistein binds 3- and 100-fold weaker, and daidzein binds 60- and 1,000-fold weaker to rat ER-β and ER-α, respectively (Boue et al. 2003; Casanova et al. 1999). These two phytoestrogens are able to elicit estrogenic responses *in vivo* (Boettger-Tong et al. 1998; Brown and Setchell 2001; Degen et al. 2002; Jefferson et al. 2002; Levy et al. 1995; Odum et al. 2001; Thigpen et al. 1997). The selective interaction of phytoestrogens with human ER-αand ER-β is similar *in vitro* to that described for the rat (Casanova et al. 1999; Kuiper et al. 1998).

Genistein is also known to have other activities, such as inhibition of different enzymes, among them tyrosine kinases (Akiyama et al. 1987), nitric oxide synthase (Duarte et al. 1997), and topoisomerase II (Okura et al. 1988), and decreasing calcium-channel activity in neurons (Potier and Rovira 1999). It also decreases lipid peroxidation (Arora et al. 1998) and diacylglycerol synthesis (Dean et al. 1989). Therefore, the multiple biologic activities of phytoestrogens raise the question of whether they have the potential to influence the outcome and/or interpretation of bioassays used to identify chemicals with estrogenic potential. In particular, questions have been raised about the presence of phytoestrogens in diets fed to animals used in bioassays designed to screen chemicals that may act as weak regulators of ERs and to screen low doses of potent regulators of ERs (Thigpen et al. 1997, 2002). One such bioassay is the uterotrophic assay, designed to evaluate both ER agonists and antagonists.

By using a version of the uterotrophic assay in the immature rat, one of the tier I screening assays recommended for detecting the estrogenic properties of endocrine-disrupting chemicals [Organisation for Economic Co-operation and Development (OECD) 2001; U.S. Environmental Protection Agency (U.S. EPA) 1998], we have identified a set of genes from the uterus and ovaries of prepubertal rats for which expression is regulated by estrogen exposure in a dose-dependent manner and which have the potential to be used as biomarkers for estrogen activity (Naciff et al. 2003). Gene expression changes induced by estrogen stimulation are more sensitive than the classical end points (i.e., uterine weight increase) for evaluating estrogenicity (Naciff et al. 2003). Given that components of the rodent diet commonly used in reproductive toxicology studies include chemicals with known estrogenic activity, understanding the influence of diet and dietary components on estrogen response is an important issue. In this study, we used gene expression profiling to evaluate the effect of two diets with different phytoestrogen content on the transcript profile of two organs that are responsive to estrogen stimulation: the uterus and the ovaries of prepubertal rats.

## Materials and Methods

### Chemicals.

17α-Ethynyl estradiol (EE) and peanut oil were obtained from Sigma Chemical Company (St. Louis, MO).

### Animals and treatments.

Fifteen-day-old female Sprague-Dawley rats were obtained (Charles River VAF/Plus; Charles River Laboratories, Raleigh, NC) in groups of 10 pups per surrogate mother. We chose this rat strain because it is commonly used in reproductive and developmental toxicity studies. The rats were acclimated to the local vivarium conditions (24°C; 12-hr light/12-hr dark cycle) for 5 days and were fed a casein-based diet (soy- and alfalfa-free diet; Purina 5K96, Purina Mills, St. Louis, MO). Starting on post-natal day (PND)20 and during the experimental phase of the protocol, all rats were singly housed in 20 × 32 × 20 cm plastic cages. To test the diet effect, there were two animal groups (*n* = 20): one group was fed a standard laboratory rodent diet (Purina 5001, Purina Mills), and the other group was maintained on the casein-based diet. The Purina 5001 diet contains phytoestrogens, mostly genistein and daidzein derived from soy and alfalfa, at levels that may have an impact on the gene expression profile (total daidzein + genistein = 0.49 mg/g; Thigpen et al. 1999), particularly in tissues regulated by estrogens such as reproductive tissues. However, those levels are not uterotrophic when evaluated by the traditional end points, uterine weight gain and increase in luminal epithelial cell height. The casein-based diet is essentially phytoestrogen free, consistently containing < 1 ppm aglycone equivalents of genistein, daidzein, and glycitein, and was fed to the four groups of animals from PND16 onward in order to remove any possible effects of the regular rodent diet (Purina 5001) previously fed to the rats by the animal supplier. All the animals were allowed free access to water and specific pelleted commercial diet (Purina 5001 or casein-based 5K96). The experimental protocol was carried out according to Procter and Gamble’s animal care approved protocols, and animals were maintained in accordance with the NIH *Guide for the Care and Use of Laboratory Animals* (Institute of Laboratory Animal Resources 1996).

Starting on PND20, each diet group was divided into two subgroups of 10 animals. One subgroup from each diet subgroup was dosed by subcutaneous injection with 0.1 μg/kg/day EE in peanut oil. This dose is not sufficient to induce a uterotrophic response in juvenile rats (Kanno et al. 2002; Naciff et al. 2003). Animals received 5 mL/kg body weight of dose solution once a day for 4 days. A 4-day dosing regime was selected to optimize detection of any effect of EE exposure at this low dose, both at the histologic level and at the gene expression level. The dose was administered between 0800 and 0900 hr each day. Controls, fed with the appropriate diet, received 5 mL/kg of peanut oil once a day for 4 days. Doses were administered on a microgram per kilogram body weight basis and adjusted daily for weight changes. Body weight (nearest 1.0 g) and the volume of the dose administered (nearest 0.1 mL) were recorded daily. The exact time of the last dose was recorded, to establish a 24-hr waiting period before tissue collection. The animals were sacrificed by CO_2_ asphyxiation 24 hr after the last dosing, on PND24. The body of the uterus, cut just above its junction with the cervix, with the ovaries attached, was carefully dissected free of adhering fat and mesentery and was weighed as a whole. Then, the ovaries were dissected free, and the uterine and ovarian wet weight was recorded. Both the uterus and ovaries were placed into RNAlater (50–100 mg/mL of solution; Ambion, Austin, TX) at room temperature.

### Histology.

Reproductive tissues from two animals in each dose group were fixed in 10% neutral buffered formalin immediately after weighing and then dehydrated and embedded in paraffin. Serial 4–5 μm cross sections were made through the ovaries, oviducts, and uterine horns, which were stained with hematoxylin and eosin. The evaluation of the morphologic changes induced by the two different diets with or without EE exposure in the uterus was performed as described previously (Naciff et al. 2003).

### Expression profiling.

We used 10 μg total RNA, extracted from uterus and ovaries from individual animals (combining only the tissues from the same animal), to prepare biotin-labeled cRNA, as previously described (Naciff et al. 2002, 2003). Labeled cRNA samples were hybridized to the Affymetrix GeneChip Test 3 Array (Affymetrix Inc., Santa Clara, CA) to assess the overall quality of each sample. After determining the target cRNA quality, we selected individual samples of pooled uteri/ovaries from five or six individual females (replicates) from each diet group, from controls, and from EE-treated subgroups (with high quality cRNA) and hybridized them to Affymetrix Rat Genome U34A high-density oligonucleotide microarrays for 16 hr. The microarrays were washed and stained by streptavidin-phycoerythrin to detect bound cRNA. The signal intensity was amplified by second staining with biotin-labeled anti-streptavidin antibody and followed by streptavidin-phycoerythrin staining. Fluorescent images were read using the Hewlett-Packard G2500A gene array scanner (Affymetrix Inc.). Affymetrix image files for the 20 chip hybridizations, and the absolute analysis results of each diet group are available from the authors upon request.

### Real-time reverse transcriptase-polymerase chain reaction.

In order to corroborate the changes in gene expression identified by the oligonucleotide microarrays, we used a real-time (kinetic) quantitative reverse transcriptase-polymerase chain reaction (QRT-PCR) approach, as previously described (Naciff et al. 2002). This approach allowed us to evaluate the “basal level” of expression of individual genes in samples derived from animals exposed to the two different diets used in our study, as well as changes induced by low-dose EE exposure (0.1 μg/kg/day). We compared the transcript level of selected genes in samples derived from animals in all experimental groups. To confirm the amplification specificity from each primer pair, the amplified PCR products were size-fractioned by electrophoresis in a 4% agarose gel in Tris borate ethylene diamine tetraacetic acid buffer and photographed after staining with ethidium bromide. Table 1 shows the nucleotide sequences for the primers used to test the indicated gene products. Preliminary experiments were done with each primer pair to determine the overall quality and specificity of the primer design. After QRT-PCR, we observed only the expected products at the correct molecular weight.

### Data analysis.

We addressed potential interindividual variability by using independent samples of each experimental group (*n* = 5 for each set) for analysis. For the uterine/ovarian weight determination, the luminal epithelial cell height, and the gene expression analysis, we compared the data from the animals fed with the casein-based diet with the data from the animals fed the normal rodent diet (Purina 5001). For gene expression analysis, scanned output files of Affymetrix micro-arrays were visually inspected for hybridization artifacts and then analyzed using Affymetrix Microarray Suite (version 5.0) and Data Mining Tool (version 3.0) software, as described by the manufacturer (Affymetrix 2002; Lockhart et al. 1996). Arrays were scaled to an average intensity of 1,500 units and analyzed independently. The Affymetrix Rat Genome U34A microarrays used in this study have 8,740 probe sets corresponding to approximately 7,000 annotated rat genes and 1,740 expressed sequence tags (ESTs).

For each transcript in the diet and dose groups, we conducted pairwise comparisons with vehicle controls fed the casein-based diet, using two-sample *t*-tests: first, we compared the two diet groups, and then we compared each treatment group with its respective diet control. We then conducted analysis of variance (ANOVA) for general diet and treatment effects on the signal value (which serves as a relative indicator of the level of expression of a transcript) and the log of the signal value. General diet effects were evaluated by ANOVA and a nonparametric test for dose–response trend, the Jonkheere-Terpstra test. Genes for which any of the tests had *p* ≤ 0.001 was taken as evidence that the expression of those genes was modified by the diet or by EE exposure. For the combined analysis of the two sets (casein-based or Purina 5001 diet), stratified nonparametric tests were conducted that were focused in detecting genes showing a diet response, or where there was a consistent treatment effect versus vehicle for the EE-treated group (0.1 μg kg/day). Here, we used linear models, with terms for both study and treatment effects, on average differences (signal values) and their log transformation, as well as stratified forms of the Wilcoxon-Mann-Whitney nonparametric statistic and a stratified form of the Jonkheere-Terpstra nonparametric statistic for diet response. Fold-change summary values for genes were calculated as a signed ratio of mean signal values (for each diet and EE-treated group compared with the appropriate control). Because fold-change values can become artificially large or undefined when mean signal values approach zero, all the values < 100 were made equal to 100 before calculating the mean signal values that are used in the fold-change calculation. All statistical analyses use the measured signal values, even if they were smaller than 100 units.

## Results

### Effect of diet on uterine/ovarian and uterine wet weight and uterine luminal epithelial cell height.

Both diets, Purina 5001 and casein-based 5K96, were well tolerated by all the animals. We observed no evidence of overt toxicity and no clinical signs of toxicity. No difference was determined in body weights between animals fed either diet (Table 2). We did not detect premature vaginal opening in any of the animals in either diet group or in animals exposed to EE. There were no differences in wet uterine weight or in absolute and relative uterine weight (Table 2) between the two diet groups, even when the animals were exposed to low doses of EE.

**Figure 1:**
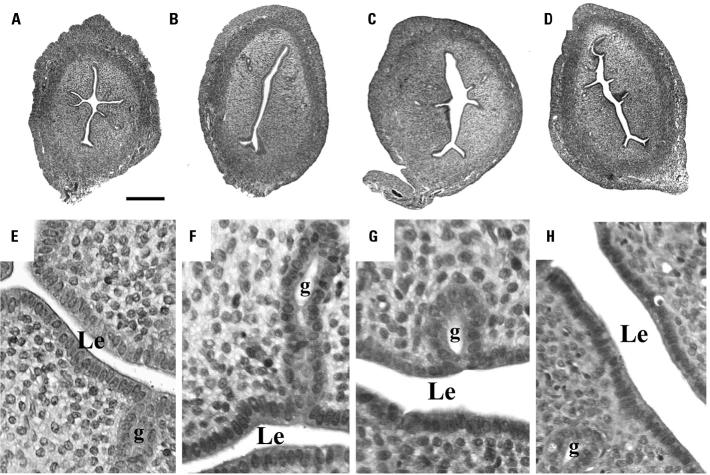
Representative uterine transversal sections from equivalent regions of vehicle-treated control immature rats (PND24; *A*, *B*, *E*, *F*) or animals treated with 0.1 μg/kg/day EE (*C*, *D*, *G*, *H*) fed with a casein-based diet (5k96; *A*, *C*, *E*, *G*) or a standard rodent diet (Purina 5001; *B*, *D*, *F*, *H*). Abbreviations: g, gland; Le, uterine lumen. See “Materials and Methods” for details. The rodent diet (*B*, *D*, *F*, *H*) containing quantifiable amounts of phytoestrogens did not have an impact on the histologic characteristics of the uterus, compared with tissues obtained from animals fed a relatively phytoestrogen-free casein-based diet (*A*, *C*, *E*, *G*). Bar = 0.08 mm for *A*–*D*; bar = 0.01 mm for *E*–*H*.

The gross anatomy of the uterus and ovaries of animals fed either diet was identical, and no signs of accumulation of fluid in the uterine lumen were noted in any of the animals. We observed no differences in uterine weight gain (wet weight) or uterine epithelial cell height (Figure 1), and we found no change in the number of uterine glands. The classical morphologic changes induced by estrogen stimulation (hypertrophy of luminal epithelial, stromal, and myometrial cells; thickening of stromal layer; and some stromal inflammatory reaction) were not observed in any of the animals exposed to the two different diets, even when exposed to 0.1 μg kg/day EE (Figure 1).

### Effect of diet on gene expression profile of the uterus/ovaries.

In order to compare the gene expression profiles induced by the different diets (different phytoestrogen content) and the EE dose tested, we compared the average value of the signal values, a relative indicator of the level of expression of a transcript, between the two groups of independent controls. We then compared the appropriate diet-control group with the respective EE group (0.1 μg/kg/day), for all the 8,740 transcripts represented on the array.

In comparing the expression profile identified in the uterus/ovaries of animals fed a casein-based diet versus the ones fed a soy/alfalfa-containing diet, we identified the expression of 29 genes that were significantly different (*p* ≤0.001). A list of those genes, along with their accession numbers, gene symbols, and the average fold changes, is shown in Table 3. The number of genes whose expression is modified by the diet’s composition is relatively small, and the average fold change on the expression of these genes affected by the rodent standard diet, compared with the casein-based diet, is relatively low in the uterus and ovaries. Although robust expression differences for specific genes can be attributed to the composition of the diet, this list does not include genes well known to be estrogen regulated, such as progesterone receptor (*PgR*), intestinal calcium-binding protein (*icabp*), and complement component 3 (*CC3*).

One hypothesis is that if the soy/alfalfa-based diet was not estrogenic on its own, perhaps it would have sufficient potency to measurably enhance the effect of a sub-uterotrophic dose of EE. Although the expression of most genes from the prepubertal uterus/ovaries that respond to estrogen exposure is not altered by the diet composition, there are some that show a variable, nonstatistically significant response. For comparison, we calculated the relative fold change induced by diet for genes that showed a clear dose response to 1–10 μg kg/day EE (Naciff et al. 2003). Presumably, those genes have the potential to represent the response to weak or low levels of estrogen stimulation (expected from the dietary phytoestrogens) and are shown in Table 4. The fold change represents the ratio of the relative expression level of each gene in tissues from animals fed Purina 5001 versus those fed the 5K96 diet (as indicated in Table 3). For comparison, in Table 4 the relative expression level of the same transcripts under EE exposure is also shown. Analyzing the effect of EE exposure on the expression of the same set of genes, comparing the relative expression level of each gene in the tissues from animals exposed to EE versus their respective controls that were fed the same diet, and taking into account that lack of statistical significance for those genes listed, the average (*n* = 5) response to EE exposure is very similar, if not equal, even for those showing a relative large fold change, regardless of the diet fed to the animals. These results suggest that the response to the diet’s composition is independent from the EE effect at this dose level of exposure. At higher doses of EE, the contribution of the dietary phytoestrogens is considered negligible because EE is a potent ER agonist able to interact with both isoforms of this receptor and with higher affinity than any of the dietary phytoestrogens (Kuiper et al. 1997). The changes induced by exposure to higher EE doses have been reported (Naciff et al. 2003). Corroboration of the microarray results by QRT-PCR for a selected group of genes is shown in Table 5. With the exception of *CC3*, which is undetectable by microarray analysis of samples derived from the animals exposed to two different diets (Table 5), the expression levels of the other genes are very similar, determined by either QRT-PCR or microarray analysis.

## Discussion

Dietary phytoestrogens, such as genistein and daidzein (abundant in soybeans and its products) and their respective glycosides (genistin and daidzin), and coumestrol (found in alfalfa), have been found to have estrogenic properties in both *in vitro* and *in vivo* (Beck et al. 2003; Boettger-Tong et al. 1998; Boue et al. 2003; Brown and Setchell 2001; Casanova et al. 1999; Degen et al. 2002; Jefferson et al. 2002; Kanno et al. 2002; Levy et al. 1995; Odum et al. 2001; Thigpen et al. 2002). However, the results of the present study showed that phytoestrogens at concentrations present in a given lot of a commercial rodent diet are not able to elicit an estrogenic response in the reproductive system of the immature rat, judged by classical end points and specific gene expression changes characteristic of estrogen exposure in estrogen-responsive target organs (uterus and ovaries). Although a number of gene expression differences were observed with the two rodent diets tested, Purina 5001 and casein-based diet (relatively high vs. low phytoestrogen content, respectively), they cannot be correlated with estrogenic activity. These gene expression changes are more likely to be caused by nutritional differences between the diets, rather than individual dietary components affecting ER pathways. Also, the traditional end points used to assess estrogenic activity, namely, uterine wet weight gain and hypertrophy of luminal epithelial cell layer, were not affected by the phytoestrogen content of the diet. It has to be stressed that we have previously identified gene expression as being far more sensitive than the classical uterotrophic response in assessing estrogenicity (Naciff et al. 2003).

We also tested whether the consumption of phytoestrogen-containing diets was sufficient to render a subuterotrophic dose regimen of EE active. To do this, we evaluated the number and type of genes whose expression is modified in the uterus/ovaries from animals exposed to 0.1 μg/kg/day EE but fed different diets. There is not a statistically different number of genes affected by components of the diet, and those genes affected by EE are the same, regardless of the diet fed to the animals. More important, the different phytoestrogen content of the diets does not modify—by either increasing or decreasing—the response of the estrogen-sensitive genes from the uterus/ovaries to low doses of a potent ER agonist; their expression changes in the same direction and magnitude as a result of EE regardless of whether the rats were fed the phytoestrogen-containing diet. Table 4 shows the transcripts that we have previously identified as being responsive to estrogen exposure, under the uterotrophic assay protocol (Naciff et al. 2003), with their relative expression level calculated by comparing the two diets. This includes genes that have an extremely robust response to estrogen exposure, such as *CC3*, *PgR*, and *icabp* (Heikaus et al. 2002; Krisinger et al. 1992; L’Horset et al. 1990; Li et al. 2002; Naciff et al. 2003). Thus, we are confident that, despite the potential effect of the phytoestrogens in the Purina 5001 diet, the transcript profile determined in the uterus and ovaries is comparable with the one determined in the animals fed the casein-based diet, and truly reflects the lack of estrogenic activity of the soy/alfalfa-based diet.

Our data corroborate the findings of the OECD (Owens et al. 2003), Wade et al. (2003), and Yamasaki et al. (2002) in the uterotrophic assay. As part of the studies conducted by the OECD validation initiative, it has been established that the phytoestrogen contents of the multiple rodent diets employed by the participant laboratories had no important effect on the sensitivity of the uterotrophic assay (Owens et al. 2003). In independent studies, Wade et al. (2003) and Yamasaki et al. (2002) reached the same conclusions by testing the effect of various phytoestrogen-containing diets in the outcome of their immature uterotrophic assays. Our findings also agree with reports on the effects of phytoestrogens on the reproductive system of other species. Foth and Cline (1998) reported that supplementing the diet of postmenopausal macaques with up to 148 mg of phytoestrogen (from soy) per day for 6 months failed to induce any proliferative effects on endometrial histology, a marker for estrogenic stimulation. Anthony et al. (1996) determined that dietary soybean isoflavones improve cardiovascular risk factors (plasma lipids, lipoproteins, and atherosclerosis) without detectable estrogenic effects in the reproductive system of peripubertal rhesus monkeys. The data presented here establish the fact that the phytoestrogens found in a regular Western diet (compared with traditional Asian diets), exemplified here as the standard rodent diet, do not elicit an estrogenic response at the histologic level or at the gene expression level. Thus, the potential benefits for humans derived from consuming a normal diet (not intentionally enriched with phytoestrogens) are not compromised by undesired estrogenic properties.

These findings demonstrate that the phytoestrogens present in a regular rodent diet do not affect the biologic response to a potent exogenous ER agonist, at the level of tissue architecture or gene expression, in prepubertal rat uterus and ovaries. From the results of the present study, it is clear that in order to elicit an estrogenic response at the gene expression level, the organism has to be exposed to higher concentrations of phytoestrogens, as has been shown in the developing female rat with pure genistein (Jefferson et al. 2002; Naciff et al. 2002). It must be stressed that the route of administration has an impact on the degree of the response; Ashby (2000) has shown that genistein gives a stronger uterotrophic response in the immature mouse when subcutaneously injected than when given orally at equivalent concentrations.

Some of the gene expression changes attributed to the composition of the diet, determined in the present study, may have an impact on the biologic response of the reproductive system (uterus/ovaries), mostly by influencing various pathways, some of which have an effect on sex hormone axis. However, none of these genes was included in the transcript profile determined for estrogens in the immature rat uterus and ovaries (Naciff et al. 2003). For example, rGrb14, the rat homologue of the human growth factor receptor, bound human Grb14 adaptor protein, a direct inhibitor of the activated insulin receptor (Bereziat et al. 2002; Kasus-Jacobi et al. 1998), whose up-regulation may result in modification of the response of the uterus/ovaries to insulin. Another gene whose expression is modified by the composition of the diet is that of the gonadotropin-releasing hormone receptor, which among other activities regulates gametogenic and hormonal functions of the gonads (Kang et al. 2003). The expression of insulin-like growth factor 1 (IGF-1) is up-regulated in the reproductive tissues of animals fed the diet with a relatively high phytoestrogen content (Table 3). IGF-1 is a critical regulator of uterine growth, and locally produced uterine IGF-1 could mediate the effects of estradiol on growth and cellular proliferation (Sato et al. 2002). The expression of the gene encoding steroid 3-α-dehydrogenase is also up-regulated by the soy/alfalfa-based diet. This enzyme, a member of the aldoketo reductase gene superfamily, is an important multifunctional oxidoreductase capable of metabolizing steroid hormones, polycyclic aromatic hydrocarbons, and prostaglandins (Huang and Luu-The 2000). Aquaporin 1 (*AQP1*) is one of the genes for which expression is down-regulated by a soy/alfalfa-based diet. This gene encodes a protein that is a member of a family of membrane channel proteins which facilitate bulk water transport and possibly other small molecules, the aquaporins. Treatment of adult ovariectomized mice with replacement steroids demonstrates an estrogen-induced shift in *AQP1* signals from the myometrium to the uterine stromal vasculature, suggesting a role in uterine fluid inhibition (Richard et al. 2003), one of the physiologic responses of the uterus to estrogen stimulation. However, the relative expression level of *AQP1* gene was not determined by Richard et al. (2003). Li et al. (1997) described a stimulatory effect of estradiol at relatively high concentrations (40 μg/kg) in the expression level of an aquaporin gene (*AQP-CHIP*) in the uterus of immature rats, although this gene was not identified as *AQP1*. However, the response of *AQP1* in the immature uterus of the rat to dietary components is actually a decrease in its expression level, opposite the effect of estrogenic stimulation.

In all, our data indicate that although there is a clear effect of the diet of the gene expression profile of the uterus/ovaries from the immature rats, this effect is subtle and cannot be correlated with the phytoestrogen content of each diet. Most of the gene transcripts represented in the microarray used in this study have an expression level that is very similar in all the animals, regardless of their diet. Further, by analyzing the expression levels of known estrogen-regulated genes (Naciff et al. 2003), we determined that there is not a significant difference in the relative expression level of any of those genes between animals exposed to Purina 5001 or casein-based diets. In addition, we found no significant changes at the transcript level for selected estrogen-regulated genes by QRT-PCR. Thus, we are confident that—despite the potential effect of the phytoestrogens in the diet of animals used in a bioassay designed to evaluate the potential estrogenic activity of a given chemical—the response to the chemical (which could be the transcript profile induced by exposure) is independent of the diet and has the potential to truly reflect estrogenic activity.

## Figures and Tables

**Table 1:** Primers used to verify the array-based gene expression changes induced by the two different diets, by QRT-PCR.

Gene name	GenBank accession no.*^a^*	Forward primer	Reverse primer	Amplicon size (bp)
Complement component 3 (*CC3*)	M29866	5′-CGTGAGCAGCACAGAAGAGA-3′	5′-CCAGGTGGTGATGGAATCTT-3′	204
Progesterone receptor (*PgR*)	L16922	5′-CATGTCAGTGGACAGATGCT-3′	5′-ACTTCAGACATCATTTCCGG-3′	428
Intestinal calcium-binding protein (*icabp*)	K00994	5′-ATCCAAACCAGCTGTCCAAG-3′	5′-TGTCGGAGCTCCTTCTTCTG-3′	196
11-β -Hydroxylsteroid dehydrogenase type 2 (*11*β *HSD*)	U22424	5′-ATGGCATTGCCTGACCTTAG-3′	5′-CTCAGTGCTCGGGGTAGAAG-3′	194
Vascular α-actin (*VaACTIN*)	X06801	5′-GACACCAGGGAGTGATGGTT-3′	5′-GTTAGCAAGGTCGGATGCTC-3′	202
Cyclophilin B	AF071225	5′-CAAGCCACTGAAGGATGTCA-3′	5′-AAAATCAGGCCTGTGGAATG-3′	239
Cytochrome P450 subfamily XVII (*Cyp17*)	M21208	5′-AAGTGGATCCTGGCTTTCCT-3′	5′-CAATGCTGGAGTCGACGTTA-3′	211
*AA924771 EST Rattus norvegicus*	AA924772	5′-TTTGCTGTGCATGGGATTTA-3′	5′-CCCTGCAGGATGTGAGAAGT-3′	202

a From GenBank (2004).

**Table 2:** Diet effect on body, uterine, and ovarian weight and luminal epithelial cell height of the juvenile (PND24) rat.

	Casein-based diet (5K96)				Purina 5001 diet			
	Body weight (g)	Ovarian weight (mg)	Uterine weight (mg)	Epithelial cell height (μm)	Body weight (g)	Ovarian weight (mg)	Uterine weight (mg)	Epithelial cell height (μm)
Peanut oil								
Mean ± SD (absolute)	68.1 ± 4.8	32.0 ± 2.6	56.1 ± 8.2	13.3 ± 1.3	70.1 ± 4.9	34.8 ± 3.3	59.6 ± 10.8	14.0 ± 2.2
Mean ± SD (relative)*^a^*		0.50 ± 0.04	0.58 ± 0.04			0.49 ± 0.07	0.56 ± 0.08	
*p*-Value*^b^*					0.05	0.18	0.41	0.26
0.1 EE (μg/kg/day)								
Mean ± SD (absolute)	68.5 ± 5.4	36.2 ± 2.1	61.9 ± 11.2	13.1 ± 1.6	71.1 ± 5.8	37.1 ± 1.8	66.4 ± 13.2	14.5 ± 1.3
Mean ± SD (relative)*^a^*		0.52 ± 0.1	0.93 ± 0.1			0.52 ± 0.1	0.93 ± 0.2	
*p*-Value*^b^*					0.05	0.14	0.36	0.18

During the experimental phase, PND20 female rats were fed with a standard laboratory rodent diet (Purina 5001) or with a soy- and alfalfa-free diet (casein-based diet, 5K96) for 5 days (from PND20 to PND24). Epithelial cell height values were obtained from tissue sections from the midregion of each uterine horn, at equivalent areas, and with clear representation of the epithelium lining the lumen along the uterus (as shown in Figure 1). Epithelial cell height was determined by obtaining five measurements from five areas from two animals for each group. These values were used to determine the mean cell height SD for each treatment group, and the corresponding *p*-value.

a Relative weight (mg/g body weight).

b Two-tailed t-test comparing 5K96 with Purina 5001, in control or treated animals; *n* = 15 for each diet group (controls) and *n* = 10 for EE-treated groups.

**Table 3:** Genes whose expression is modified by exposure to diet in the uterus/ovaries of the immature rat.

GenBank accession no.*^a^*	Gene name	Gene symbol	Average fold change*^b^*	*p*-Value*^c^*
X67948	Aquaporin 1 (aquaporin channel forming integral protein)	*AQP1*	1.6	0.000159
U56839	Purinergic receptor P2Y, G-protein coupled 2	*P2ry2*	1.4	0.000448
AF017756	GSK-3beta interacting protein rAxin	*Axin*	1.4	0.000130
AA859529	Diacylglycerol acyltransferase	*Dgat*	1.3	0.000470
L06096	Inositol 1,4,5-triphosphate receptor 3	*Itpr3*	1.3	0.000420
U90887	Arginase type II	*Arg2*	1.3	0.000728
U78977	ATPase, class II, type 9A	*Atp9a*	1.3	0.000022
AA892562	EST196365, high homology to nucleolar protein NAP57 and dyskeratosis congenita 1, dyskerin	*Dkc1*	1.3	0.000747
AI639534	ESTs, similar to properdin (factor P)		1.3	0.000446
AI231213	ESTs, high homology to kangai 1 (suppression of tumorigenicity 6), prostate	*Kai1*	1.2	0.000561
D10874	Vacuolar H(+)-transporting ATPase,		1.2	0.000865
X56133	Mitochondrial H+-ATP synthase alpha subunit	*Atp5a1*	−1.1	0.000854
D13417	Transcription factor HES-1 homolog of hairy and enhancer of split 1, (*Drosophila*)	*Hes1*	−1.2	0.000045
Z71925	Polymerase (RNA) II (DNA directed) polypeptide G	*Polr2g*	−1.2	0.000379
AA818487	ESTs, high homology to cyclophilin B	*Ppib*	−1.2	0.000253
AI112237	ESTs, moderately similar to JE0384 NADH dehydrogenase		−1.2	0.000192
AA818858	Peptidylprolyl isomerase A (cyclophilin A)	*Ppia*	−1.3	0.000943
AA686579	ESTs, similar to ubiquitin-like protein SMT3C precursor		−1.3	0.000954
U64705	Protein synthesis initiation factor 4AII gene and E3 small nucleolar RNA gene		−1.3	0.000405
S69316	GRP94/endoplasmin (5 and 3 regions)		−1.3	0.000120
M15481	Insulin-like growth factor 1	*IGF-1*	−1.3	0.000068
S69315	GRP94/endoplasmin (5 and 3 regions)		−1.4	0.000174
D17310	3-alpha-Hydroxysteroid dehydrogenase (3-alpha-HSD)		−1.4	0.000397
X67859	Autoantigen or Sjogren syndrome antigen B	*Ssb*	−1.4	0.000103
AA685903	ESTs, similar to glucose regulated protein, 94 kDa	*GRP94*	−1.5	0.000878
S68578	Gonadotropin-releasing hormone receptor	*Grhr*	−1.5	0.000322
AI009141	EST203592, *Rattus norvegicus*		−1.8	0.000468
AF076619	Growth factor receptor bound protein 14 or molecular adapter rGrb14 (Grb14), an inhibitor of insulin actions	*Grb14*	−2.1	0.000158

a From GenBank (2004).

b The average fold change was determined by comparing the average signal value of the indicated transcripts obtained from the uterus/ovaries from five females fed with the casein-based diet (5K96) versus the average signal value obtained from the same tissues from five females fed the standard rodent diet (Purina 5001).

c Transcripts listed are those showing a robust response to the different diet (*p* < 0.001) using the stratified form of the Jonkheere-Terpstra nonparametric statistic to identify the diet response.

**Table 4:** Diet effect on genes whose expression is modified by exposure to of 0.1 μg/kg EE in the uterus/ovaries of the immature rat.

			Average fold change*^b^*		
GenBank accession no.*^a^*	Gene name	Gene symbol	Purina 5001/5K96	EE vs. control, 5K96	EE vs. control, Purina 5001
M29866	Complement component 3	*CC3*	A	14.7	7.0
Y08358	Eotaxin or small inducible cytokine A11	*Scya11*	A	2.7	2.9
AI013389	ESTs, similar to calcium-binding protein, intestinal, vitamin D-dependent	*Calb3*	2.0	2.4	1.9
K00994	Intestinal calcium-binding protein	*icabp*	1.1	5.2	2.1
U49062	CD24 antigen	*Cd24*	1.1	1.3	1.2
L14004	Polymeric immunoglobulin receptor	*pigr*	1.5	1.4	1.1
AA859661	ESTs, similar to glutaminyl-peptide cyclotransferase precursor		A	2.1	1.7
M57718	Cytochrome P450 IV A1	*CYP4A1*	A	A	A
U22424	Hydroxysteroid dehydrogenase, 11-βtype 2	*Hsd11b2*	1.0	2.2	1.6
L07114	Apolipoprotein B editing protein	*Apobec1*	A	A	A
S79730	Opioid receptor-like ORL1 receptor	Oprl1	1.2	1.3	1.4
M88469	*f*-Spondin	*Sponf*	1.7	1.7	1.1
X66845	Dynein, cytoplasmic, intermediate chain 1	*Dncic1*	A	A	A
L46593	Small proline-rich protein gene	*Sprr*	2.4	2.5	−1.0
L00191	Fibronectin, encoding three mRNAs, exons 1, 2, 3	*fn*	−1.2	1.6	1.2
M22323	Gamma-enteric smooth muscle actin	*Actg2*	1.4	1.5	1.3
D15069	Adrenomedullin	*Adm*	1.9	2.4	1.2
AA893870	EST197673 *Rattus norvegicus*		A	1.7	2.0
AI232078	Transforming growth factor-β (TGF-β) masking protein	*Ltbp1*	−1.1	1.2	1.1
U82612	Fibronectin (fn-1) gene	*fn-1*	1.5	1.6	1.1
X05834	Fibronectin (fn-3) gene	*fn-3*	1.0	1.4	1.2
L00382	Skeletal muscle β-tropomyosin and fibroblast tropomyosin 1	*tpm1*	1.2	1.7	1.3
AA800908	EST190405 *Rattus norvegicus*		1.2	1.6	1.4
M25758	Phosphatidylinositol transfer protein	*Pitpn*	1.1	1.3	1.3
AA799773	ESTs, *Rattus norvegicus*		1.3	1.3	1.2
AA892829	EST, similar to mouse bifunctional 3’-phosphoadenosine (PPS1)	*PPS1*	1.2	1.3	1.1
AB010963	Potassium large conductance calcium-activated channel	*Kcnmb1*	1.2	1.4	1.2
AF083269	Actin-related protein complex 1b	*Arpc1b*	1.0	1.3	−1.0
AA891760	EST195563 *Rattus norvegicus*		A	A	A
AJ005394	Collagen α1 type V	*Col5a1*	−1.1	1.6	1.2
L11930	Cyclase-associated protein homologue	*Cap1*	1.1	1.3	1.2
X07467	Glucose-6-phosphate dehydrogenase	*G6pd*	1.2	1.2	−1.1
U26310	Tensin	*Tns*	1.2	1.3	1.1
AA891542	EST195345 *Rattus norvegicus*, similar to mouse heat shock protein hsp40-3	*Dnajb5*	1.3	1.3	1.1
U44948	Cysteine-rich protein 2 or smooth muscle cell LIM protein (SmLIM)	*Csrp2*	−1.1	−1.6	−1.4
S61868	Ryudocan or heparan sulfate proteoglycan core protein or syndecan-4	*SDC4*	−1.2	−1.5	−1.1
L41254	Corticosteroid-induced protein or FXYD domain-containing ion transport regulator 4	*Fxyd4*	−1.1	−1.4	−1.1
AF023087	Nerve growth factor induced factor A, or early growth response 1	*Egr1*	−1.5	−1.7	1.1
U07181	Lactate dehydrogenase B	*Ldhb*	−1.3	−1.2	−1.1
X89225	Solute carrier family 3, member 2	*Slc3a2*	−1.3	−1.5	−1.3
AF054826	Vesicle-associated membrane protein 5	*Vamp5*	−1.2	−1.8	−1.2
X75253	Phosphatidylethanolamine binding protein	*Pbp*	−1.2	−1.4	−1.1
AA924772	ESTs, similar to metallothionein 3	*Mt3*	A	A	A
AA894027	EST197830 *Rattus norvegicus*		A	A	A
AA894030	EST197833 *Rattus norvegicus*		A	A	A
AA946532	ESTs, similar to ATP-binding cassette, sub-family D (ALD), member 3	*Abcd3*	−1.3	−1.4	−1.3
M32754	Inhibin α-subunit	*Inha*	−1.3	−1.6	1.0
AA874794	ESTs, similar to nerve growth factor receptor (TNFRSF16) associated protein 1	*Ngfrap1*	1.0	−1.4	−1.2
M21060	Superoxide dismutase 1, soluble	*Sod1*	−1.2	−1.2	−1.2
X08056	Guanidinoacetate methyltransferase	*GAMT*	−1.2	−1.3	−1.0
D00729	δ 3, δ2-enoyl-CoA isomerase		−1.2	−1.2	−1.3
U90829	APP-binding protein 1	*Appbp1*	−1.2	−1.6	−1.1
AI170613	ESTs, similar to heat shock 10 kDa protein 1	*Hspe1*	−1.2	−1.3	−1.6
D63761	Adrenodoxin reductase	*Fdxr*	−1.1	−1.5	−1.1
D78303	Splicing factor YT521-B	*YT521*	−1.1	−1.2	−1.2
L48060	Prolactin receptor	*Prlr*	1.0	−1.3	−1.2
AA849036	ESTs, similar to guanylate cyclase 1, soluble, α-3	*Gucy1a3*	−1.2	−1.3	−1.1
M33648	Mitochondrial 3-hydroxy-3-methylglutaryl-CoA synthase	*HMGCS2*	−1.2	−1.6	−1.3
E05646	Phosphatidylethanolamine binding protein	*Pbp*	−1.2	−1.3	−1.2
AA858520	ESTs, similar to follistatin	*Fst*	−1.3	−1.5	−1.1
L02842	Follicle-stimulating hormone receptor	*FSHR*	A	A	A
X04229	Glutathione-*S*-transferase, μ type 1 (Yb1)	*Gstm1*	−1.2	−1.4	−1.2
L23148	Inhibitor of DNA binding 1, helix-loop-helix protein	*Id1*	1.0	−1.1	1.0
D63761	Adrenodoxin reductase	*Fdxr*	1.0	−1.4	−1.2
AF076619	Growth factor receptor bound protein 14	*Grb14*	−1.5	−1.8	−1.3
M33648	Mitochondrial 3-hydroxy-3-methylglutaryl-CoA synthase		−1.3	−2.0	−1.6
AA858520	Follistatin	*Fst*	−1.3	−1.4	−1.3
X62660	Glutathione transferase subunit 8		−1.2	−1.5	−1.3
AI175776	EST219344 *Rattus norvegicus*		−1.2	−1.7	−1.4
J03914	Glutathione-*S-*transferase, μ type 2 (Yb2)	*Gstm2*	−1.2	−1.2	−1.1
J02592	Glutathione-*S*-transferase, μ type 2 (Yb2)	*Gstm2*	−1.1	−1.3	−1.2
S59525	Gonadotropin-releasing hormone receptor	*grhr*	−1.1	−1.8	−1.4
M36453	Inhibin α	*Inha*	−1.2	−1.6	−1.6
X54793	Heat shock protein 60 (liver)	*Hsp60*	−1.3	−1.3	−1.6
AA858640	ESTs		−1.2	−1.4	−1.4
L19998	Minoxidil sulfotransferase	*PST-1*	−1.2	−1.4	−1.4
X78848	Glutathione-*S*-transferase, αtype (Ya)	*Gsta1*	−1.2	−1.5	−1.4
AF001898	Aldehyde dehydrogenase 1, subfamily A1	*Aldh1a1*	1.1	−1.5	−1.5
X97754	Hydroxysteroid dehydrogenase 17β, type 1	*Hsd17b1*	−1.5	−2.3	−1.6
AF000942	Inhibitor of DNA binding 3, dominant negative helix-loop-helix	*Id3*	−1.2	−1.3	−1.1
AI171268	EST217223 *Rattus norvegicus*, identical to inhibitor of DNA binding 3, dominant negative helix-loop-helix	*Id3*	−1.4	−1.4	−1.1
D84336	Delta-like homolog (*Drosophila*), a novel member of the epidermal growth factor (EGF)-like family of proteins	*Dlk1*	A	A	A
S63167	3 β*-*Hydroxysteroid dehydrogenase isomerase type II	*HSD3B2*	−1.1	−1.4	−1.6
M12492	Type II cAMP-dependent protein kinase regulatory subunit	*prkar2a*	−1.3	−1.9	−1.8
S72505	Glutathione *S*-transferase Yc1 subunit		−1.2	−1.5	−1.6
AA874919	Mismatch repair protein	*Msh2*	−1.1	−1.5	−1.1
M14656	Sialoprotein (osteopontin)	*Spp1*	1.0	−3.5	−2.4
X01115	SVS-protein F, or seminal vesicle secretion 5	*Svs5*	−1.4	−3.9	1.1
M21208	Cytochrome P450, subfamily XVII	*Cyp17*	1.1	−2.4	−2.2

A, absent (undetectable by Microarray Suite 5.0; Affymetrix). Transcripts listed are those previously reported to show a robust response to graded doses of EE in the uterotrophic assay (*p* < 0.001) (Naciff et al. 2003); *n* = 5 per group.

a From GenBank (2004).

b The average fold change is the ratio of the relative expression level of each gene in uterus/ovaries from animals fed Purina 5001 versus those fed 5K96 diet (*n* = 5 per group).

**Table 5:** Selected gene expression changes verified by QRT-PCR.

	*CC3*		*icabp*		*11*β *HSD*		*PgR*		*EST AA924772*		*VaACTIN*		*Cyp17*		*Cyclo B*	
Treatment	Q	M	Q	M	Q	M	Q	M	Q	M	Q	M	Q	M	Q	M
Vehicle in Purina 5001 vs. 5K96	1.0	A	1.2	1.1	1.2	1.0	1.1	1.1	−1.1	A	1.2	1.1	−1.2	1.1	1.1	1.0
0.1 EE μg vs. control in 5K96	18.7	14.7	3.7	5.2	2.7	2.2	1.8	1.6	−1.5	A	1.1	1.0	−3.5	−2.4	1.0	1.1
0.1 EE μg vs. control in Purina 5001	21.2	7.0	3.3	2.1	2.9	1.6	2.2	1.5	−1.4	A	1.1	1.1	−3.4	−2.2	1.1	1.0

Abbreviations: *11*β*HSD*, 11-β-hydroxylsteroid dehydrogenase type 2 gene; A, absent (undetectable by Microarray Suite 5.0; Affymetrix); *cyclo B*, cyclophilin B gene; *Cyp17*, cytochrome P450 subfamily XVII gene; M, microarray-derived fold change; Q, QRT-PCR–derived fold change; *VaACTIN,* vascular α-actin gene. The relative fold change is the ratio of the relative expression level of each gene in uterus/ovaries from animals fed Purina 5001 versus those fed 5K96 diet. The microarray-derived fold change and the QRT-PCR–derived fold change were determined as described in “Materials and Methods,” using the same amount of total RNA derived from three independent animals, in duplicate. These genes were chosen on the basis of their response to estrogenic stimulation in the uterotrophic assay (Naciff et al. 2003); we also included two control genes, *cyclo B* and *VaACTIN*.
